# Genome-Wide Differential Expression Profiling of Ovarian circRNAs Associated With Litter Size in Pigs

**DOI:** 10.3389/fgene.2019.01010

**Published:** 2019-11-15

**Authors:** Gaoxiao Xu, Huifang Zhang, Xiao Li, Jianhong Hu, Gongshe Yang, Shiduo Sun

**Affiliations:** ^1^Key Laboratory of Animal Genetics, Breeding and Reproduction of Shaanxi Province, College of Animal Sciences and Technologies, Northwest A&F University, Yangling, China; ^2^Teaching and Research Section of Biotechnology, Nanning University, Nanning, China

**Keywords:** circRNA, ovary, litter size, pig, miRNA

## Abstract

Circular RNAs (circRNAs) have been emerging as an important regulator in mammalian reproduction *via* acting as miRNA sponges. However, the circRNAs in porcine ovaries related with litter size remains largely unknown. In this study, porcine ovaries with smaller or larger litter size (LLS) were subjected to high-throughput RNA sequencing. In total, 38,722 circRNAs were identified, of which 1,291 circRNAs were commonly expressed in all samples. There were 56 circRNAs significantly down-regulated and 54 circRNAs up-regulated in LLS pig (|log2 (fold change) | > 1, FDR < 0.05). Bioinformatics predicted that most of circRNAs harbored miRNA binding sites, and the expression patterns of circRNAs and their putative binding miRNAs were validated by qPCR. Moreover, the expression of circ-*TCP11*/miR-183 was significantly reversely correlated and their direct interaction was confirmed by dual-luciferase assay. Our study indicates that circRNAs may play potential effects on modulating porcine litter size.

## Introduction

Increasing litter size has been a global goal for pig breeders and producers, and larger litter size plus shorter farrowing intervals are desperately expected to expand piglets per sow per year, which is the predominant force to boost economic success of sow husbandry (Zak et al., 2017; Kemp et al., 2018). Ovary is an important reproductive organ in females and goes through a series of biological processes during each estrous cycle. Sow prolificacy is tightly modulated by the complex transcriptional network involving coding and non-coding genes in ovaries (Zhang et al., 2015; Huang et al., 2016; Tang et al., 2018).

Covalently closed circular RNAs (circRNAs) are emerging as a novel class of modulators for gene expression (Li et al., 2018). Now, accumulating work has shed light on the critical roles of circRNAs in gonadal development and reproduction performance in many species (Quan and Li 2018). Next-generation sequencing has revealed that endogenous circRNAs are generally expressed in various kinds of porcine tissues in a spatio-temporally specific manner, including ovaries (Liang et al., 2017). Recent studies revealed that human ovary-derived circRNAs are involved in ovarian aging (Cai et al., 2018), thus we investigated whether circRNAs’ profile differs in sows with different litter sizes.

In this study, a total of six ovaries were selected from multiparous sows with intact prolificacy records, and high-throughput sequencing technology coupled with bioinformatic tools were employed to uncover litter-size-related circRNAs, providing potential candidate loci that may be informative for future pig breeding programs.

## Materials and Methods

### Ethics Statement and Sample Collection

This study was approved by the Animal Care and Use Committee in Northwest A&F University (No. 2018-019). The ovaries in our study were collected from sows 4 days after the fourth delivery picked from a commercial sow piggery in Hanshiwei Food Ltd., Co. (Dahua, Guangxi, China), which is negative for PRRSV (porcine reproductive and respiratory syndrome virus) and PCV (porcine circovirus). For RNA-seq, three ovaries were sampled from each group small and large litter size (8.48 ± 0.53 piglets/litter in small litter size and 16.19 ± 0.43 piglets/litter in large litter size). In RT-qPCR assay, more ovaries were sampled (8.78 ± 1.75 piglets/litter *vs.* 14.83 ± 1.61 piglets/litter, n = 12). All sows were slaughtered in a standard slaughterhouse in Xinyouxian Livestock Ltd., Co., (Xining, Guangxi, China), and the left ovaries were quickly taken and frozen in liquid nitrogen.

### RNA-Seq Assay

The frozen ovary tissues were homogenized in TRIzol™ reagent (Invitrogen, Carlsbad, CA, USA), and each sample was quantified using ND-1000 Nanodrop (Thermo Fisher, Wilmington, DE, USA). RNA integrity number (RIN) was analyzed with Agilent 2200 (Agilent, Palo Alto, CA, USA), and RNAs with RIN >7.0 were used for RNA-seq analysis.

The total RNA samples (3 μg) were treated with Epicenter Ribo-Zero rRNA removal kit (Illumina, San Diego, CA, USA) to remove ribosomal RNA (rRNA) before cDNA library construction, and then ribosome depleted RNAs were fragmented into 150–200 nt by incubation with divalent cations at 94°C for 8 min. The cleaved RNA fragments were reverse-transcribed into first- and second-strand cDNA according to the description of TruSeq RNA LT/HT sample preparation kit (Illumina, USA). Briefly, the cDNA was treated with End-It DNA End Repair Kit to repair the ends, then modified with Klenow to add an A at the 3′ end, and finally ligated to indexed adapters. The ligated cDNA products were purified and treated with uracil DNA glycosylase to remove the second-strand cDNA. Purified first-strand cDNA was enriched by 13–16 cycles of PCR amplification. The final cDNA libraries were evaluated by Bioanalyzer 2200 (Agilent, Santa Clara, CA) and subjected to sequencing by HiSeq 2000 (Illumina, USA).

miRNA libraries were constructed by Ion Total RNA-Seq Kit v2.0 (Life Technologies), and the sizes were selected by PAGE gel and processed for miRNA sequencing.

### Bioinformatic Analysis

Raw sequencing data were tested by performing FAST-QC (http://www.bioinformatics.babraham.ac.uk/projects/fastqc/) and evaluation metrics including quality scores, distribution of nucleotides, GC content, k-mer frequency, and others. Low-quality bases and N bases were trimmed from the reads by NGSQCToolkit (v2.3.3), and high-quality clean reads were obtained for subsequent analysis. Clean reads were mapped to the reference genome (Sscrofa 11.1 assembly, http://genome.ucsc.edu) using Hisat2 software.

CIRI was used to identify circRNAs. The alignment results (SAM format) were scanned to search paired chiastic clipping and paired-end mapping signals, as well as GT-AG splicing signals. All the sequences with junction sites were realigned to reference genome using dynamic programming algorithm to ensure the reliability of the putative circRNAs. The total number of reads spanning back-spliced junctions was used as an absolute measure of circRNA abundance.

DEseq2 was used to explore the differentially expressed mRNA among different groups, and the criteria were set as |log2 (fold change) | > 1, FDR < 0.05. EdgeRSeq was used to explore the differentially expressed circRNA and miRNA between groups, with cutoff of |log2 (fold change) | > 1, FDR < 0.05. Gene ontology (GO) function (http://www.geneontology.org/) and Kyoto Encyclopedia of Genes and Genomes (KEGG, http://www.genome.jp/kegg) pathway of genes of target were annotated.

### CeRNA Network Construction

The potential miRNA–circRNA interactions were predicted by miRanda (http://miranda.org.uk/) and RNAhybrid2 (http://bibiserv.techfak.uni-bielefeld.de/rnahybrid). The correlation between the expression levels of circRNA and miRNA was calculated with SPSS Pearson correlation assay.

### RT-qPCR Verification

Total RNA was purified using TRIzol™ reagent (Invitrogen). An aliquot of 2 μg total RNA was taken from each sample and reverse transcripted by random primers (TakaRa, Otsu, Japan). For miRNA analysis, specific reverse transcription primers and procedures were used. Real-time PCR reaction (95°C 30 s, then 95°C 5 s, 60°C 30 s for 40 cycles, following 70°C 10 min for elongation) was performed in triplicate using the One-step SYBY PrimeScript RT-PCR kit (TakaRa) on a Bio-Rad iQ5™ system (Bio-Rad, Berkeley, CA, USA). The expressions of circRNA and miRNA were normalized to that of *ACTB* and *U6* small RNA, respectively. The primer sequences for qPCR are shown in **Table 1**.

**Table 1:** Sequences of primers used in this study.

Primer list	Sequence (5′→3′)	Product length (bp)
circ-*ERBIN*	F: TTCGACATCCCCAGACATCC R: CAGACATCCGAGACGGAGAA	218
circ-*SNTB2*	F: CAACAATGGAGACCCGTCCT R: TCCTTGGTGCTGTTCTGGTG	267
circ-*TCP1*	F: GGGACCTTAAACGCATTGCTA R: GGTCCAGTTTTTCAGGGTCTGT	147
circ-*KMT2A*	F: AGGAGAACGCAGGCACTTTG R: GGAGGAGGTTCACTGTTGCT	261
circ-*LOC397451*	F: GCTTGCATTGAAAACGGGTCTC R: TCCATTACAGGCAGGACAGTG	261
circ-*NUP98*	F: AGCACAGGGACCAGTCTTTTC R: AGGCTTCCAGTATTGTTGCTG	248
circ-*SENP2*	F: GGGGAAGAGCAAAGTCATGGA R: TCCGTGTGCCATTACAAGCA	294
circ-*CCDC85A*	F: CTGCTAGACTTGACCAGCGTT R: CAAATGTGGGCCAATGGTGAT	180
circ-*CCAR1*	F: CCAACATCAGCAGCCCTTGT R: TGCTGCAATCCGAGTATCCC	218
*ACTB*	F: GGACTTCGAGCAGGAGATGG R: AGGAAGGAGGGCTGGAAGAG	134

### Dual Luciferase Assay

A ∼400 bp fragment of circRNA containing the putative miRNA binding site was synthesized by General Biosystems (Chuzhou, Anhui, China) and inserted into psiCHECK™-2 vector (Promega, Madison, WI, USA) to construct psi-circRNA plasmids. Then the psi-circRNA constructs were co-transfected with their corresponding miRNAs (RiboBio, Guangzhou, China) into 293T cells using Lipofectin™ 2000 (Thermo Fisher Scientific, Waltham, MA, USA), and the luciferase activity was detected by Dual-Luciferase^®^ Reporter Assay System (Promega) 24 h post transfection.

### Statistical Analysis

Data were processed with SPSS 19.0 software, and results were presented as mean ± SEM. Significant differences were assessed by unpaired Student’s *t*-test and *p* < 0.05 was defined as statistical significance.

## Results

### Overview of CircRNAs in Porcine Ovary

**Figure 1:**
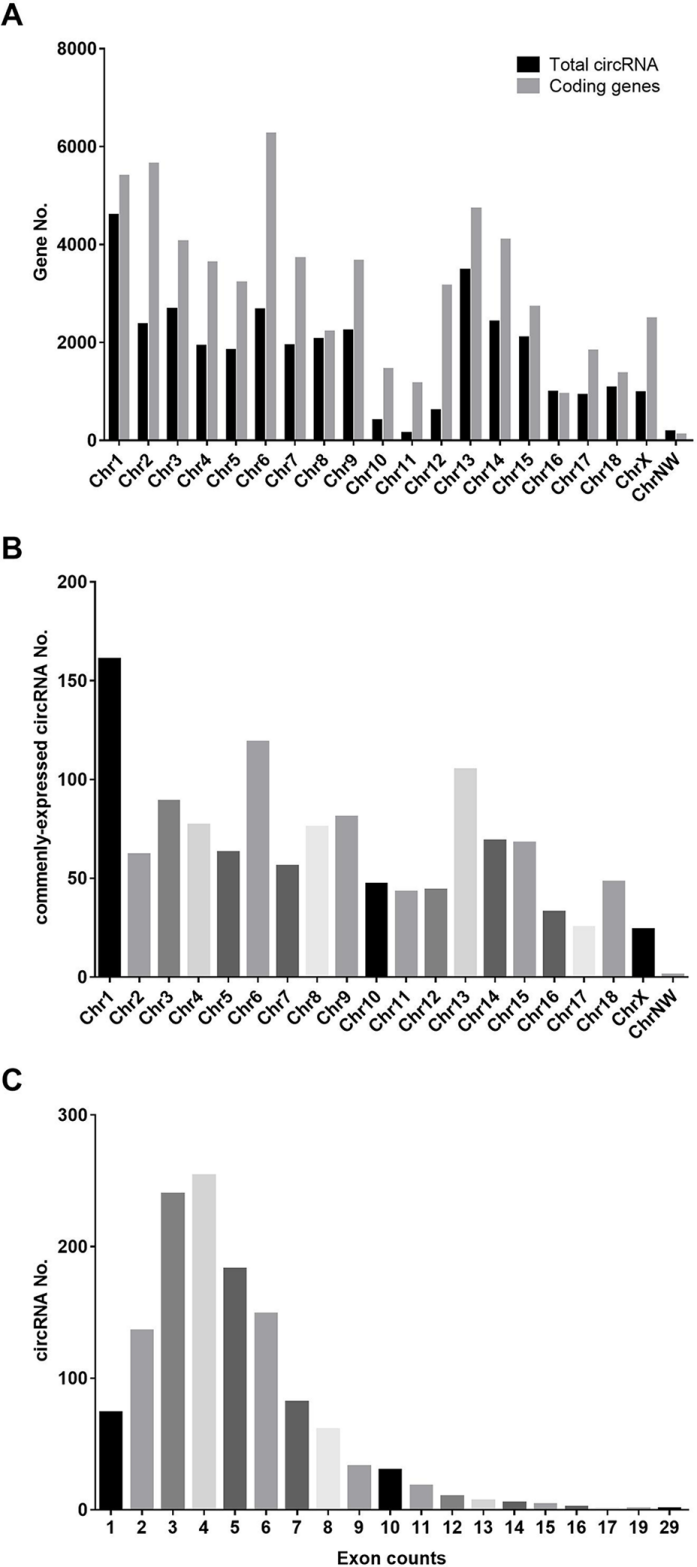
Distribution of predicted circRNAs in our study. **(A)** The distribution of annotated mRNAs on the genome. **(B)** The distribution of commenly-expressed circRNAs on the genome. **(C)** The exon numbers of commenly-expressed circRNAs in our study.

Three ovaries in each group (small litter size *vs.* large litter size) were subjected to RNA sequencing, and a total of 38,722 circRNAs were predicted, which were widely distributed across all chromosomes (**Figures 1A, B**). However, only 1,291 circRNAs were expressed in all samples (**Figure 1C**).

### Differential CircRNA Expression Profiles in Pigs Differing Litter Size

**Figure 2:**
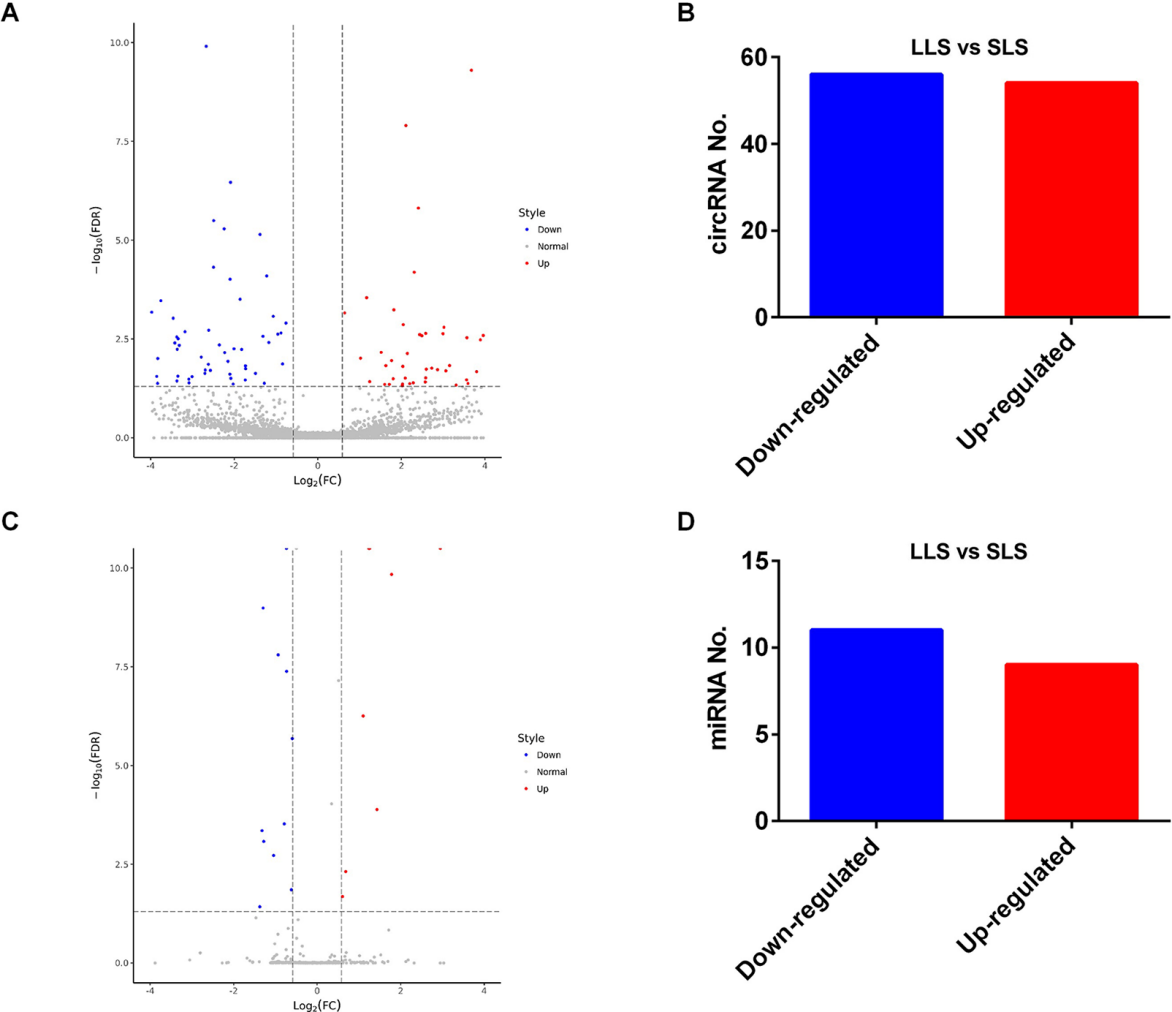
Differentially expressed circRNAs and miRNAs between SLS and LLS pigs. **(A)** The volcano plot of differentially-expressed circRNAs in LLS and SLS ovaries. **(B)** The numbers of down- and up-regulated circRNAs in LLS ovaries compared with SLS. **(C)** The volcano plot of differentially-expressed miRNAs in LLS and SLS ovaries. **(D)** The numbers of down- and up-regulated miRNAs in LLS ovaries compared with SLS.

In our study, 110 circRNAs [56 down-regulated and 54 up-regulated in larger litter size (LLS)] (**Figure 2 A, B**) and 20 miRNAs [11 down-regulated and 9 up-regulated in smaller litter size (SLS)] were identified by RNA-seq (**Figure 2 C, D**).

**Figure 3:**
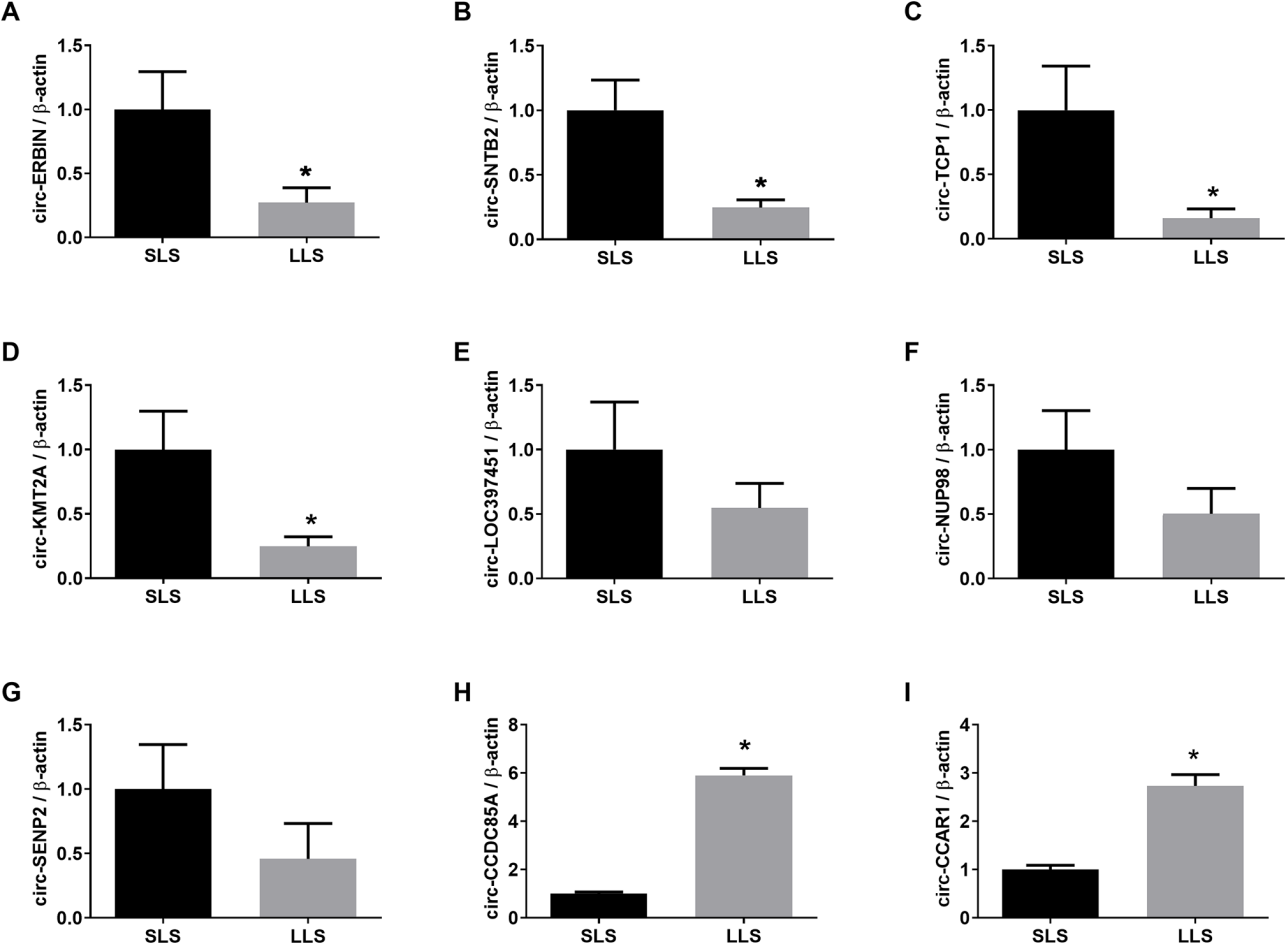
Validation of differentially expressed circRNAs by qPCR. The expression of circ-ERBIN **(A)**, circ-SNTB2 **(B)**, circ-TCP1 **(C)**, circ-KMT2A **(D)**, circ-LOC397451 **(E)**, circ-NUP98 **(F)**, circ-SENP2 **(G)**, circ-CCDC85A **(H)** and circ-CCAR1 **(I)** in SLS and LLS ovaries. *presents p < 0.05.

Given the high variability between samples in RNA-seq, the sample pool was expanded to 12 per group, and a total of 24 ovaries were used when confirming the differentially expressed circRNAs and miRNAs revealed above using RT-PCR. Based on the expanded sample size, RT-PCR assay uncovered that circ-*ERBN*, circ-*SNTB2*, circ-*TCP1*, and circ-*KMT2A* were significantly higher expressed in porcine ovaries with SLS, while circ-*CCDC85A* and circ-*CCAR1* were significantly higher expressed in porcine ovaries with LLS (**Figure 3**).

**Figure 4:**
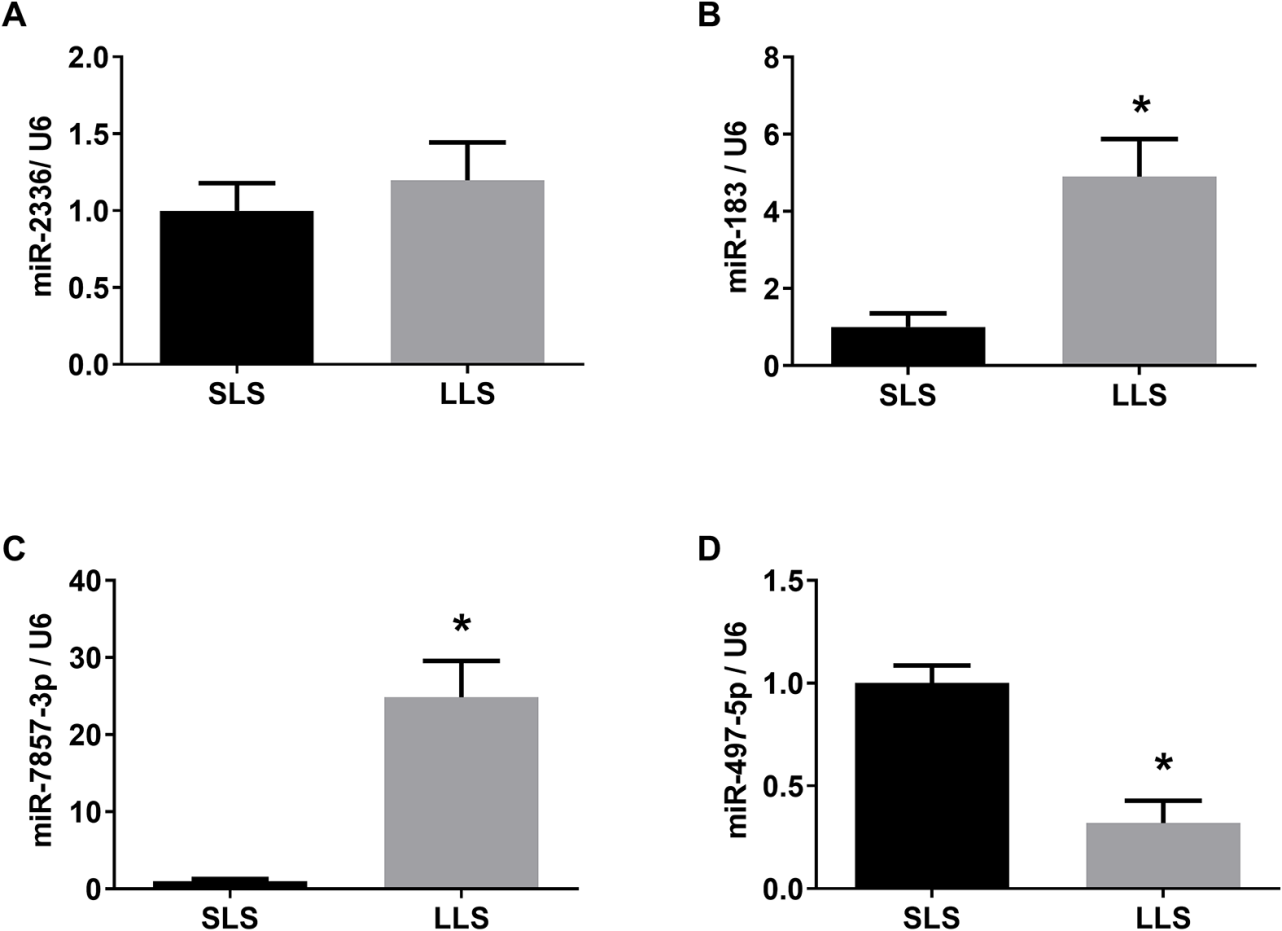
Validation of differentially expressed miRNAs by qPCR. The expression of miR-2336 **(A)**, miR-183 **(B)**, miR-7857 **(C)** and miR-497-5p **(D)** in SLS and LLS ovaries. *presents p < 0.05.

Regarding the differentially expressed miRNAs, the levels of miR-183 and miR-7857-3p were significantly lower in the SLS group while miR-497-5p were significantly lower expressed in pigs with LLS, which were consistent with high-throughput sequencing(**Figure 4**).

### Identification of CircRNA–MiRNA Axis

**Figure 5:**
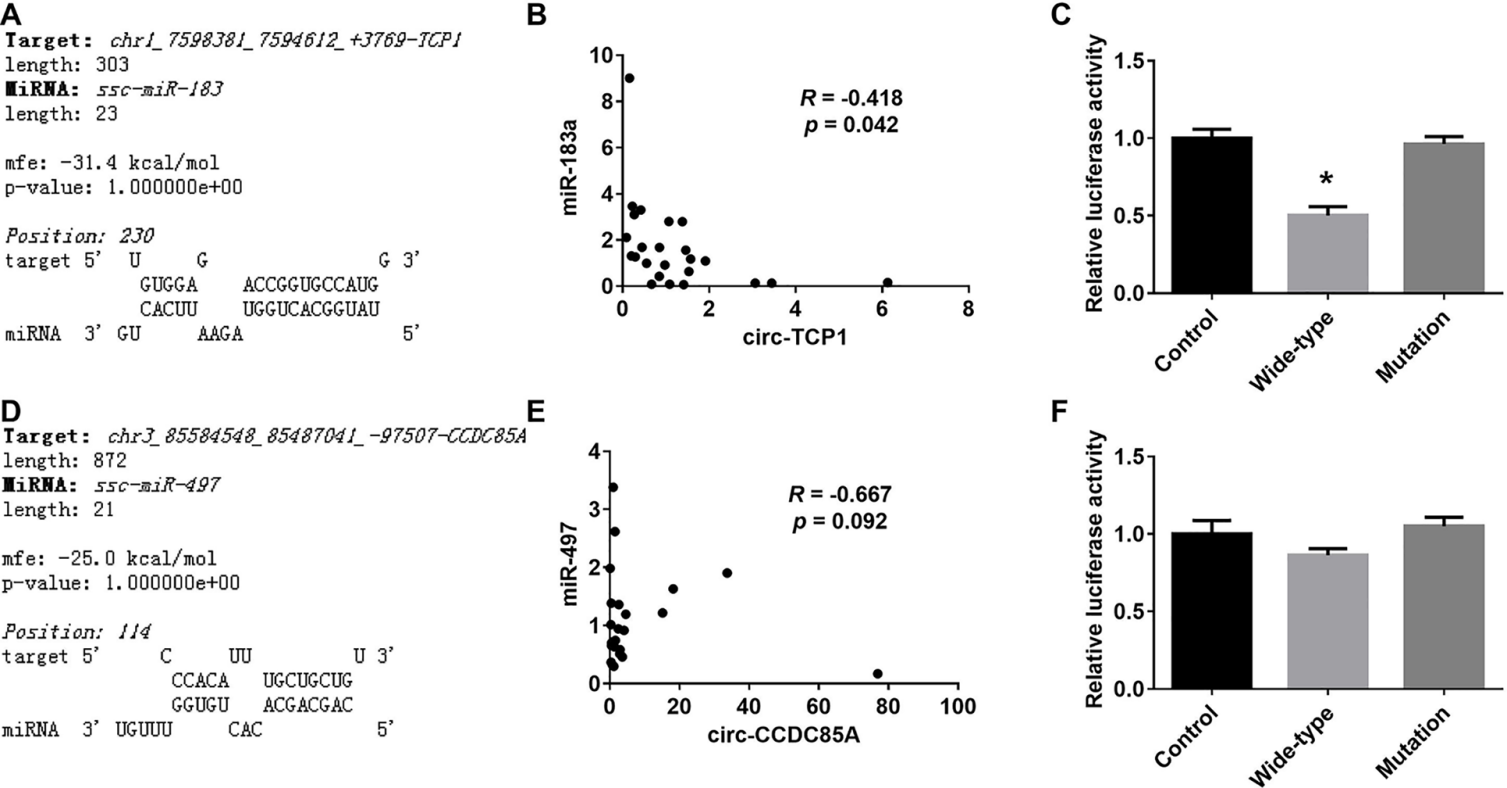
Validation of the interaction between the differentially expressed miRNAs and circRNAs. **(A)** The putative binding site of miR-183 on circ-TCP1 predicted by RNAhybrid. **(B)** The correlation of the expression levels between miR-183 and circ-TCP1 in ovaries. **(C)** The relative luciferase activity of reporter constructs containing wild-type or mutant miR-183-binding sites from circ-TCP1 following cotransfection with control or miR-183 mimics. **(D)** The putative binding site of miR-497 on circ-CCDC85A predicted by RNAhybrid. **(E)** The correlation of the expression levels between miR-497 and circ-CCDC85A in ovaries. **(F)** The relative luciferase activity of reporter constructs containing wild-type or mutant miR-497-binding sites from circ-CCDC85A following cotransfection with control or miR-497 mimics. *presents p < 0.05.

Among the differentially expressed circRNAs and miRNAs detected above, RNAhybrid analysis revealed that miR-183 was predicted to interact with circ-*TCP1*, miR-497 with circ-*CCDC85A*. Besides, the expression of miR-183 was reversely correlated with circ-*TCP1*, and a similar tendency was observed between miR-497 and circ-*CCDC85A*. Meanwhile, dual-luciferase reporter assay has shown that miR-183 could directly bind to circ-*TCP1*, while the direct interaction between miR-497 and circ-*CCDC85A* was not detected in this assay (**Figure 5**).

### Function Analysis of miR-183

**Figure 6:**
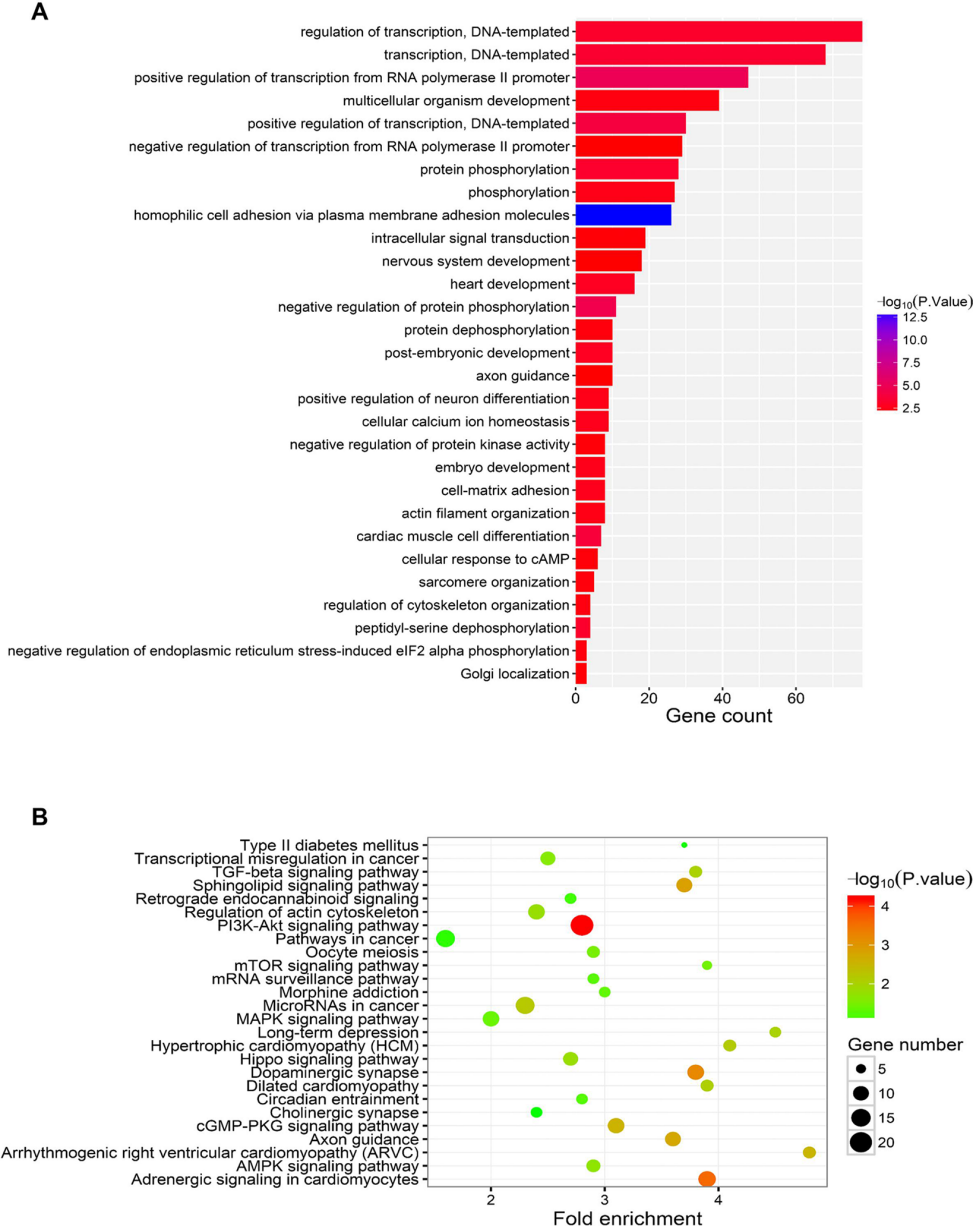
Functional analysis of miR-183. **(A)** The KEGG pathway analysis of miR-183 target genes. **(B)** The GO enrichment analysis of miR-183 target genes.

TargetScan and MiRDB were used to predict the potential targets of miR-183, and the common genes presented by these two strategies were subjected to KEGG and GO analysis. KEGG showed that miR-183 might modulate DNA-templated and RNA PolII-mediated transcription (**Figure 6A**). GO enrichment assay indicated that miR-183 might be tightly related with PI3K-Akt signaling activity (**Figure 6B**).

## Discussion

The aim of the current study was to identify potential circRNAs related with swine fertility. Even RNA-seq only screened out a total of 1,291 exon-derived circRNAs that expressed in each ovary in our study; there were still several circRNAs that differentially expressed in ovaries with small and large litter size. To overcome the deficiency of great individual variation, the expressions of circRNAs of interest were further validated by RT-qPCR on larger-scale samples. Of note, significantly more circ-*TCP1* was detected in porcine ovaries with SLS, and we focus on circ-*TCP1* in the subsequent study.

One of the well-documented pathways of circRNAs is to competitively bind to functional miRNAs, known as competing endogenous RNAs (ceRNAs) (Li et al., 2018). Here, circ-*TCP1*, derived from exons 7 and 8 of porcine *TCP1* (T-complex protein 1 subunit alpha) gene, was significantly lower expressed in ovaries with LLS. *TCP1* gene encodes a molecular chaperone that is a member of the chaperonin containing TCP1 complex (CCT), also known as the TCP1 ring complex (TRiC), which folds various proteins, including actin and tubulin (Sternlicht et al., 1993). Currently, there are no reports about the role of *TCP1* in ovary. However, *CCT6A*, the zeta subunit of CCT, was shown to be expressed in chicken granulosa cells, indicating an important role in folic growth (Wei et al., 2013). Here, circ-*TCP1* was predicted to absorb miR-183 by online RNAhybrid software. Moreover, miR-183 presented a significantly reverse profile with circ-*TCP1*, and the interaction between circ-*TCP1* and miR-183 was further confirmed by luciferase activity assay. Collectively, our data indicated that the circ-*TCP1*–miR-183 axis might be involved in the biological processes related with litter size.

MiR-183 belongs to the highly conserved miR-183-96-182 cluster, which have been shown to be associated with female fertility (Zhang et al., 2019a). Members of miR-183-96-182 cluster are known to target the 3′-UTR of *FOXO1*, an important transcription factor for follicle-stimulating hormone responsive genes in ovarian granulosa cells of rodents (Herndon et al., 2016). *FOXO1* and miR-183-96-182 cluster have been also shown to be associated with bovine ovarian follicle development (Zielak-Steciwko and Evans 2016). MiR-183 is highly expressed in ovarian cancer cells (Wang et al., 2014; Chen et al., 2016) and down-regulation of miR-183 markedly represses cell proliferation and promotes apoptosis *via* targeting SMAD family member 4 (*Smad4*) (Zhou et al., 2019). In our study, KEGG and GO assay suggested that miR-183 might be associated with gene transcription, especially related with PI3K-Akt signaling. Genome-wide analysis revealed that genes related with PI3K-Akt activity were changed during different follicular stages in the ovaries of Duroc pigs (Liu et al., 2018), and PI3K-Akt activity was significantly inhibited when cell growth of porcine ovarian granulosa was impaired by extracellular stimuli (Wang et al., 2019; Zhang et al., 2019b). Altered PI3K-Akt signaling was also reported to contribute to impeded 17β-estradiol secretion in ovary cells (Wu et al., 2017). However, the molecular mechanism of miR-183-PI3K-Akt axis in ovary and their effects on litter size requires further investigation.

## Conclusion

In our study, genome-wide identification of exon-derived circRNAs in porcine ovaries was performed by RNA-seq, and many of which were differently expressed in ovaries with variant litter sizes. Furthermore, most exonic circRNAs harbored miRNA binding sites, and circ-*TCP1*-miR-183 axis might be associated with swine litter size.

## Data Availability Statement

The data generated in this study has been uploaded to NCBI and can be found under accession number: GSE136592

## Ethics Statement

This study was approved by Animal Care and Use Committee in Northwest A&F University.

## Author Contributions

GX conducted the study and drafted the manuscript. HZ and XL assisted t in ovaries sampling and data analysis. JH, GY gave critical comments about experiment design and manuscript drafting. SS supervised the experiment.

## Funding

This work was supported by the National Key Technology R and D Program of China (2015BAD03B01-10).

## Conflict of Interest

The authors declare that the research was conducted in the absence of any commercial or financial relationships that could be construed as a potential conflict of interest.
